# The added value of longitudinal black-blood cardiovascular magnetic resonance angiography in the cross sectional identification of carotid atherosclerotic ulceration

**DOI:** 10.1186/1532-429X-11-31

**Published:** 2009-08-18

**Authors:** Wei Yu, Hunter R Underhill, Marina S Ferguson, Daniel S Hippe, Thomas S Hatsukami, Chun Yuan, Baocheng Chu

**Affiliations:** 1 Department of Radiology, University of Washington, Seattle, WA, USA; 2 Department of Radiology, Beijing Anzhen Hospital, Capital Medical University, Beijing, PR China; 3 Department of Surgery, Vascular Surgery Division, University of Washington, Seattle, WA, USA

## Abstract

**Background:**

Carotid atherosclerotic ulceration is a significant source of stroke. This study evaluates the efficacy of adding longitudinal black-blood (BB) cardiovascular magnetic resonance (CMR) angiography to cross-sectional CMR images in the identification of carotid atherosclerotic ulceration.

**Methods:**

Thirty-two subjects (30 males and two females with ages between 48 and 83 years) scheduled for carotid endarterectomy were imaged on a 1.5T GE Signa scanner using multisequence [3D time-of-flight, T1, proton density, T2, contrast enhanced T1], cross-sectional CMR images and longitudinal BB CMR angiography (0.625 × 0.625 mm/pixel). Two rounds of review (round 1: cross-sectional CMR images alone and round 2: cross-sectional CMR images plus longitudinal BB CMR angiography) were conducted for the presence and volume measurements of ulceration. Ulceration was defined as a distinct depression into the plaque containing blood flow signal on cross-sectional CMR and longitudinal BB CMR angiography.

**Results:**

Of the 32 plaques examined by histology, 17 contained 21 ulcers. Using the longitudinal BB CMR angiography sequence in addition to the cross-sectional CMR images in round 2, the sensitivity improved to 80% for ulcers of at least 6 mm^3 ^in volume by histology and 52.4% for all ulcers, compared to 30% and 23.8% in round 1, respectively. There was a slight decline in specificity from 88.2% to 82.3%, though both the positive and negative predictive values increased modestly from 71.4% to 78.6% and from 48.4% to 58.3%, respectively.

**Conclusion:**

The addition of longitudinal BB CMR angiography to multisequence cross-sectional CMR images increases accuracy in the identification of carotid atherosclerotic ulceration.

## Background

Stroke is the third most common cause of death and a leading cause of serious, long-term disability worldwide [1]. Extracranial carotid disease accounts for 20–30% of such ischemic events [2]. Carotid atherosclerotic ulceration is a significant contributing source of cerebral ischemic incidents or stroke, along with other plaque features such as intraplaque hemorrhage and thrombosis [3-10].

Although angiography, specifically digital subtraction angiography (DSA), time-of-flight cardiovascular magnetic resonance (CMR) angiography, or computed tomography angiography (CTA), has become the standard approach to carotid artery disease assessment, these modalities are limited by their inability to show the outer wall of the vessel. Black-blood CMR can depict the actual plaque size by imaging both the lumen and the outer wall of an artery. Studies comparing in vivo black-blood CMR with corresponding endarterectomy specimens confirm the ability of black-blood CMR to accurately visualize atheroma size [11-13]. On the other hand, time-of-flight CMR angiography yields lower estimates of luminal narrowing compared to black-blood CMR angiography [13,14], and underestimates the presence of high-risk plaque features [15]. These findings highlight the need for an improved clinical vessel wall imaging method to accurately grade the severity and stage of carotid atherosclerosis [15].

Previous studies have shown good sensitivity and specificity in the identification of carotid atherosclerotic plaque components and the condition of the fibrous cap overlying the necrotic core using multisequence high-spatial-resolution cross-sectional CMR imaging [11,16-20]. Identification of fibrous cap rupture is highly associated with recent transient ischemic attack or stroke [17]. Furthermore, studies have demonstrated that detection of carotid atherosclerotic ulceration is possible using these multisequence cross-sectional CMR images [21,22]. However, cross-sectional CMR images may suffer from partial volume effects due to the intrinsic morphological changes of the carotid bulb [23-25]. Flow artifacts that mimic plaques [23] and may potentially confound the clear detection of ulcerations occur frequently in this region. The addition of longitudinal black-blood (BB) CMR angiography may reduce errors in classification due to partial volume effects by producing images with greater resolution in the long-axis view of the carotid artery. Longitudinal BB CMR angiography is therefore advocated for pre-operative assessment of the long-axis extent of advanced lesions and for determining the precise location of the carotid bifurcation [26].

In this study, we hypothesize that adding the longitudinal BB CMR angiography to multisequence high-spatial-resolution cross-sectional CMR images can improve the ability of CMR in the characterization of carotid atherosclerotic ulceration.

## Materials and methods

### Subjects

From 1998 to 2004, 58 patients (50 males and 8 females, ages between 48 and 83, and median age 69 years) scheduled for carotid endarterectomy at the University of Washington Medical Center or VA Puget Sound Health Care System underwent preoperative carotid CMR after obtaining informed consent. All subjects either experienced a transient ischemic attack, amaurosis fugax, or stroke within 6 months of their surgery and had >50% carotid stenosis by duplex ultrasound or were asymptomatic with >80% carotid stenosis.

Twenty-two subjects were excluded from this study because there was no BB CMR angiography available, three subjects were excluded due to poor image quality [20], and one subject was excluded because the histology specimen was too damaged to review. Of the original 58 subjects, 32 (30 males and two females with ages 48–83) with both BB CMR angiography and histology of their excised plaque were analyzed.

### CMR acquisition

The carotid CMR scans were performed within one week prior to carotid endarterectomy. Patients were imaged with a 1.5T GE Signa scanner (Horizon EchoSpeed, General Electric Healthcare, Milwaukee, USA) using a custom designed 4-element, phased-array carotid surface coil to improve the signal-to-noise performance [27].

**Figure 1:**
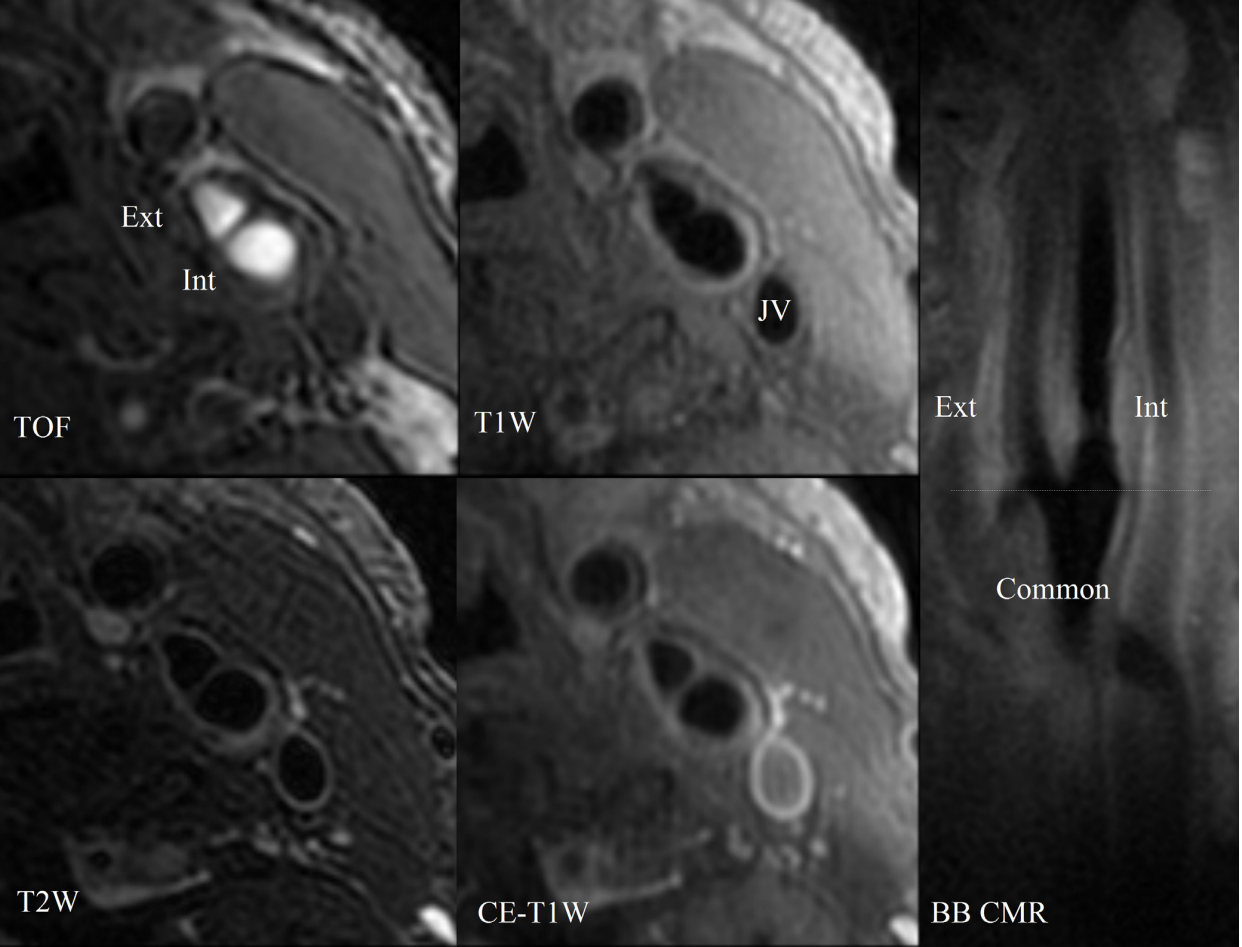
**Example of carotid multisequence cross-sectional CMR images and a longitudinal black-blood CMR (BB CMR) angiography**. Four contrast-weighted CMR images [3-dimensional time-of-flight (TOF), T1-weighted (T1W), T2-weighted (T2W), and contrast-enhanced T1-weighted (CE-T1W)] are matched using the carotid bifurcation (dashed line) seen on the longitudinal BB CMR angiography as the landmark. Proton density weighted image is not shown. Int = internal carotid artery. Ext = external carotid artery. Common = common carotid artery. JV = jugular vein.

The carotid CMR scans were performed using a standard imaging protocol, which includes longitudinal BB CMR angiography and five contrast weighted cross-sectional images. The longitudinal BB CMR angiography (Figure 1) was acquired centered on the carotid bifurcation identified by a 2-dimensional time-of-flight (2D TOF) on the operative side. The scan used a multi-slice double inversion recovery 2D fast spin echo (FSE) sequence [repetition time (TR), 1800 ms; effective echo time (TE), 9.5 ms; field of view (FOV), 160 × 160 mm; matrix, 256 × 256 (in-plane resolution: 0.625 × 0.625 mm/pixel); thickness, 2 mm; Section gap, 0 mm; and number of excitation (NEX), 2]. A total of six images were obtained per carotid artery [26].

Five contrast weighted cross-sectional images [3-dimensional time-of-flight (3D TOF), proton density (PD), T2-, and pre- and post-contrast enhanced T1-weighted images] of the carotid arteries were acquired using the carotid bifurcation as a landmark. The imaging protocol consisted of the following sequences: 1) 2D pre-contrast quadruple inversion recovery (QIR) T1-weighted FSE (TR, 800 ms; TI 1/TI 2, 375/125 ms; effective TE, 11 ms) [28]; 2) 2D cardiac gated PD weighted FSE (TR, 3 R-R intervals; effective TE, 17.9 ms); 3) 2D cardiac gated T2-weighted FSE (TR, 3 R-R intervals; effective TE, 68 ms) [29]; 4) 3D TOF CMR angiography (TR, 23 ms; TE, 3.6 ms; flip angle, 25 degrees); and 5) 2D post-contrast QIR T1-weighted FSE with the same parameters as for the pre-contrast T1-weighted FSE sequence. A gadolinium-based contrast agent (Omniscan, GE Healthcare) was administered intravenously at a dose of 0.1 mmol/kg body weight for post-contrast QIR T1-weighted FSE sequence (Figure 1).

All cross-sectional images were obtained with a field of view of 160 × 160 mm, matrix size of 256 × 256 (in-plane resolution: 0.625 × 0.625 mm/pixel), section thickness of 2 mm, and NEX of 2. Section gap was 0 mm for pre- and post-contrast enhanced T1-, PD, and T2-weighted images, and -1 mm for 3D TOF (1 mm overlapping between adjacent images). The coverage of each carotid artery was 24 mm (12 images) for pre- and post-contrast enhanced T1-, 36 mm (18 images) for PD and T2-weighted images, and 40 mm (40 images) for 3D TOF source images.

### CMR matching and review

**Figure 2:**
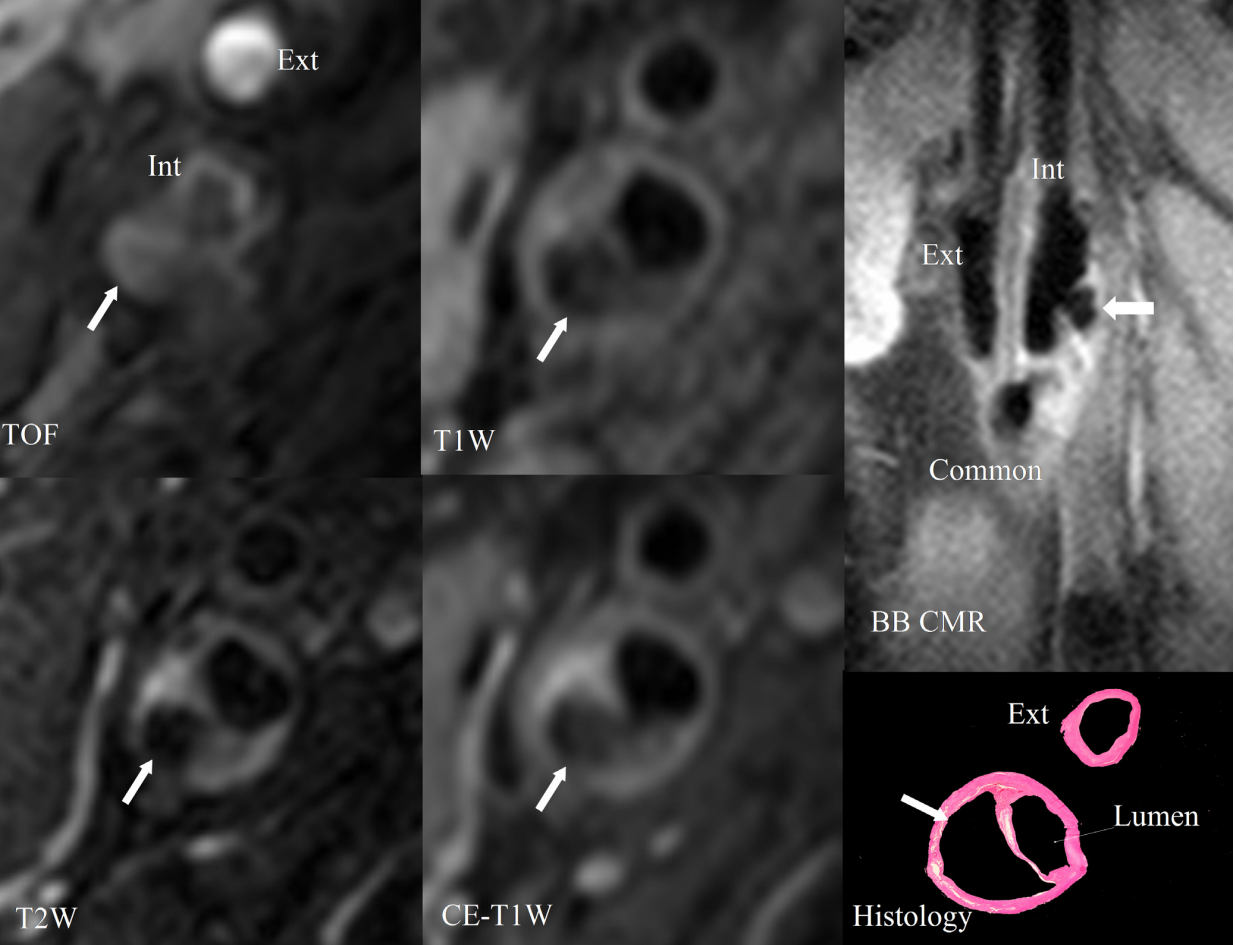
**Ulcer identification using multicontrast, cross-sectional CMR images and longitudinal black-blood CMR (BB CMR) angiography**. The ulcer (arrows), with a disorganized blood flow signal pattern shown on the cross-sectional CMR images, is well demarcated on the longitudinal BB CMR angiography and confirmed by corresponding histology. Proton density weighted image is not shown. Int = internal carotid artery. Ext = external carotid artery. Common = common carotid artery.

The five contrast weighted cross-sectional CMR images were matched using the carotid bifurcation as a landmark. CMR images were then analyzed in two separate rounds of review. The interval between the reviews was 103 days. The first round used only the five cross-sectional contrast weighted CMR images. All CMR slices were evaluated for the presence of ulcer by one reviewer (W.Y.) and were peer reviewed by another reviewer (H.U.). Ulcer was defined as a surface disruption with a depression into the plaque containing a disorganized blood flow signal on the five contrast-weighted cross-sectional CMR images. In the second round, BB CMR angiography series were included and the first round of CMR review was revisited. The reviewer (W.Y.) modified the results of the first round if her interpretation changed with the addition of BB CMR angiography. The second round was also peer reviewed by H.U. In the second round of review, the same ulcer definition was applied to both the cross-sectional slices and BB CMR angiography. If disagreement occurred, consensus opinion was adopted following discussion between the two reviewers. Both reviewers were blinded to histology during the two rounds of review (Figure 2).

During both rounds, the area of each ulcer was measured from every slice on which it was identified using custom-built software package (CASCADE) [30].

### Histology review

A reviewer (M.S.F; blinded to CMR data) examined all histology sections. A slice was classified as having ulceration if either of the following was true: 1) The luminal surface (endothelial cells) was disrupted and the length along the luminal surface and depth into the wall (both measured on the cross-sections) were each at least 0.5 mm; 2) A disruption on the luminal surface appeared to be a continuation from an adjacent slice already classified as having ulcer, regardless of the length and depth. These criteria were designed to be as close to the CMR criteria as possible while excluding likely processing artifacts, such as shrinkage and distortion of the specimens during processing.

The areas of the disrupted surfaces were measured using the custom software (CASCADE).

### CMR and histology matching

**Figure 3:**
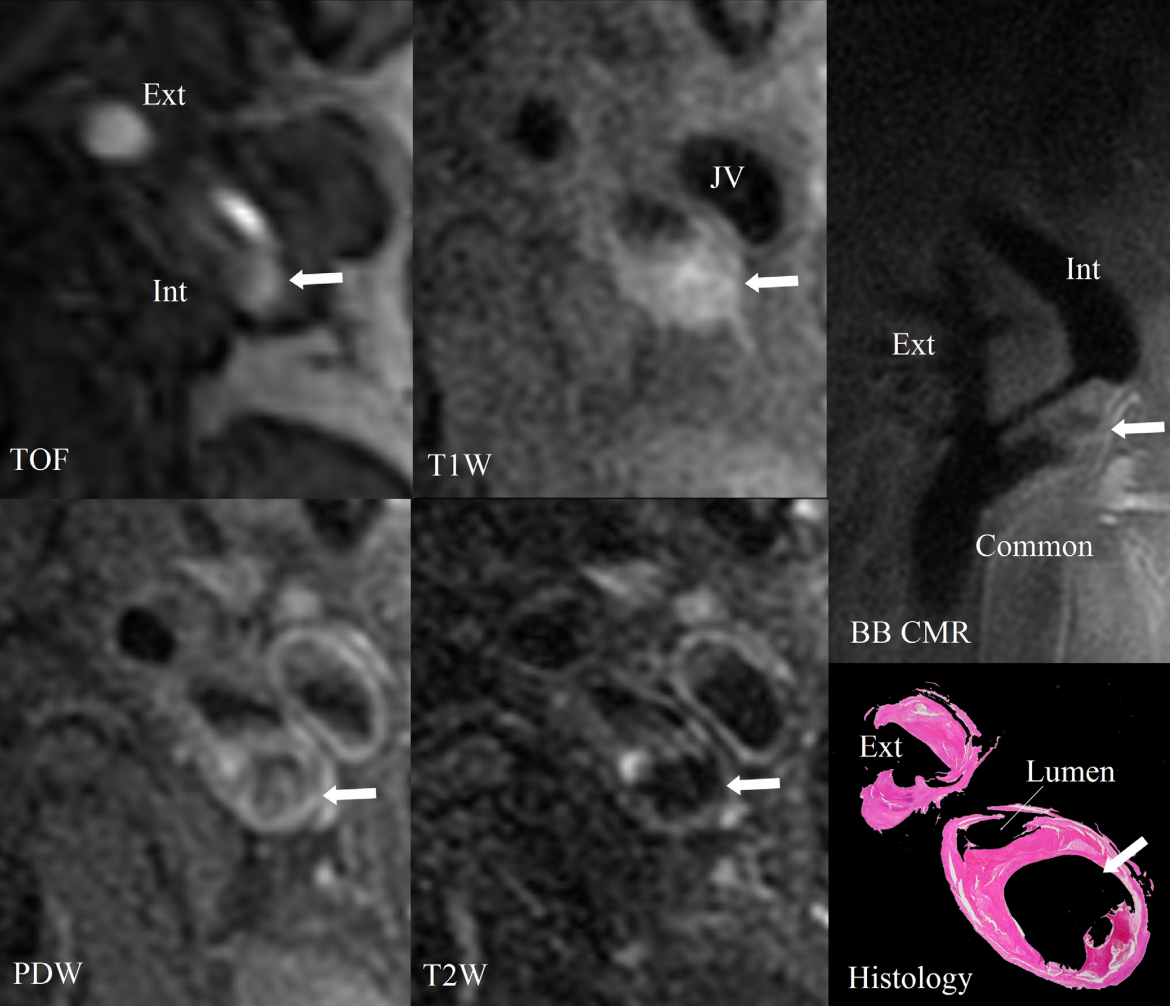
**Example of partial volume and flow disturbance in a large penetrating ulcer**. The cross-sectional CMR images alone could not verify the presence of the ulcer in the left carotid artery. The signal pattern of the ulcer resembles the signal pattern of intraplaque hemorrhage (arrows). However, the longitudinal black-blood CMR (BB CMR) angiography shows clear separation between the very narrow lumen and the penetrating ulcer due to the improvement of spatial resolution in the longitudinal direction. The flow artifact with this ulcer can be easily identified. Corresponding histology confirms the presence of the ulcer. CE-T1W is not shown. PDW = proton density weighted. Int = internal carotid artery. Ext = external carotid artery. Common = common carotid artery. JV = jugular vein.

The contrast weighted cross-sectional CMR images were matched with histology using the carotid bifurcation (Figures 2 and 3) and gross morphological features such as lumen size and shape, wall size and shape, and plaque configuration. At each matched location histology provided the definitive information. In the case where more than one histology section matched a given CMR slice (as histology is collected at every millimeter while CMR images are collected at every 2 millimeters) the final histology ulcer classification was positive if any of the sections were positive.

### Statistical analysis

For both the CMR and histology results, adjacent slices identifying the same ulcer were aggregated into single ulcer observations (the unit of analysis). The volume of each ulcer was computed by multiplying the ulcer area by the distance to the next slice distal.

CMR ulcer classification was evaluated using histology as the gold standard. A true positive was defined as when an ulcer by CMR and an ulcer by histology had at least one location in common. A true negative was defined as when both CMR and histology identified no ulcer in the entire plaque. False positives (negatives) were defined as when an ulcer by CMR (histology) had no locations in common with any ulcers by histology (CMR).

The sensitivity, specificity, positive predictive value and negative predictive value were calculated using the number of true and false positives and negatives of ulcers. As ulcer size was expected to be related to detection performance, sensitivity was plotted versus volume. For each point on the plot, the sensitivity was recalculated using only ulcers greater than the given volume.

The Mann-Whitney rank test was used to assess differences between independent groups of observations, and Spearman's rank correlation was used to assess association between the ulcer volume measurements by CMR and histology. All p-values are two-tailed and all analyses were done using statistical software R2.6.2.

## Results

Of the 32 plaques examined by histology, 17 contained a total of 21 ulcers (14 plaques had a single ulcer, 2 had two ulcers, and 1 had three ulcers). After matching with the CMR images, of the 21 ulcers, 8 occurred on only one cross-sectional CMR slice and 9 occurred on three or more slices.

During round 1 (cross sectional review only), 5 true ulcers were detected. Another six ulcers were detected by adding the BB CMR angiography in round 2. The ulcers detected were significantly larger by histology (median 20 mm^3 ^vs. 2.8 mm^3^, p = 0.034).

**Figure 4:**
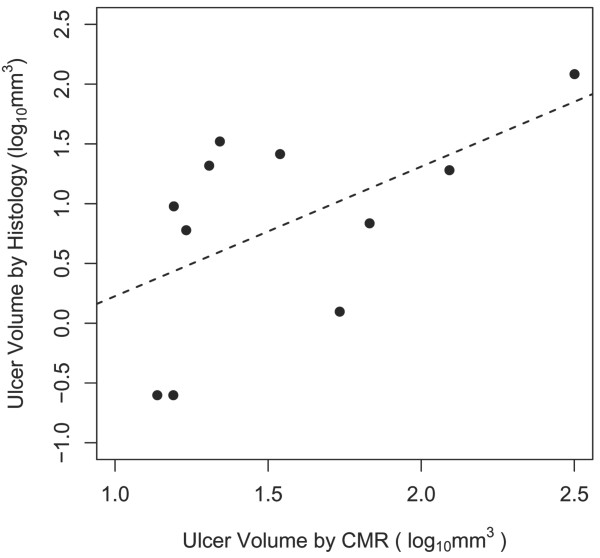
**Scatter plots of the ulcer volume on log scale measured by CMR and histology**. Only ulcers where histology and CMR agreed in round 2 review (cross-sectional CMR images plus BB CMR angiography) were included (n = 11). Spearmen's rank correlation was calculated as r = 0.60 (p = 0.053).

The Spearman's rank correlation between the volume measurements of the ulcers detected by both CMR and histology in round 2 was 0.60 (n = 11, p = 0.053) (Figure 4).

**Figure 5:**
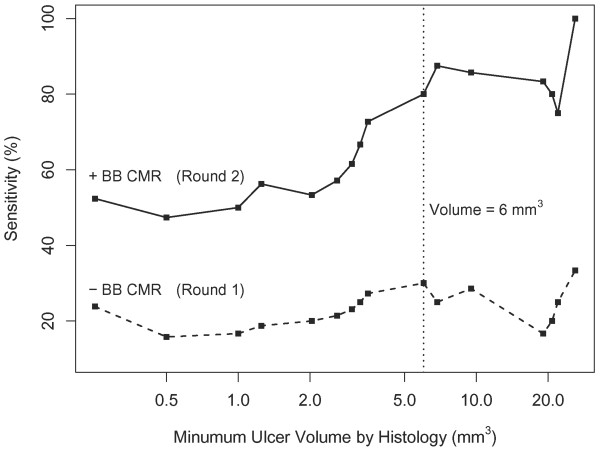
**Sensitivity of ulcer identification by CMR with regard to ulcer volume by histology**. The sensitivity was calculated at each point in the Figure using only ulcers with the given volume or greater. The sensitivity was uniformly higher in round 2 review (cross-sectional CMR images plus BB CMR angiography), and in particular was notably higher for larger ulcers. Sensitivity did not appear to depend on size in round 1 review (cross-sectional CMR images alone).

The sensitivity from rounds 1 and 2 were graphed versus ulcer volume by histology, where at each point on the graph, only ulcers of that size or greater were included (Figure 5). In round 2, the sensitivity was 52.4% for all ulcers (n = 21) and 80.0% for ulcers at least 6 mm^3 ^(n = 10). By contrast, the sensitivity from round 1 did not show a clear trend versus size. It was 23.8% and 30.0% for all ulcers and ulcers at least 6 mm^3^, respectively.

Due to an additional false positive, the specificity decreased slightly from 88.2% in round 1 to 82.3% in round 2. However, both the positive and negative predictive values increased modestly in round 2 (from 71.4% to 78.6% and from 48.4% to 58.3%, respectively). When considering only ulcers at least 6 mm^3 ^by histology, the negative predictive value improved from 73.1% to 90.0%.

**Figure 6:**
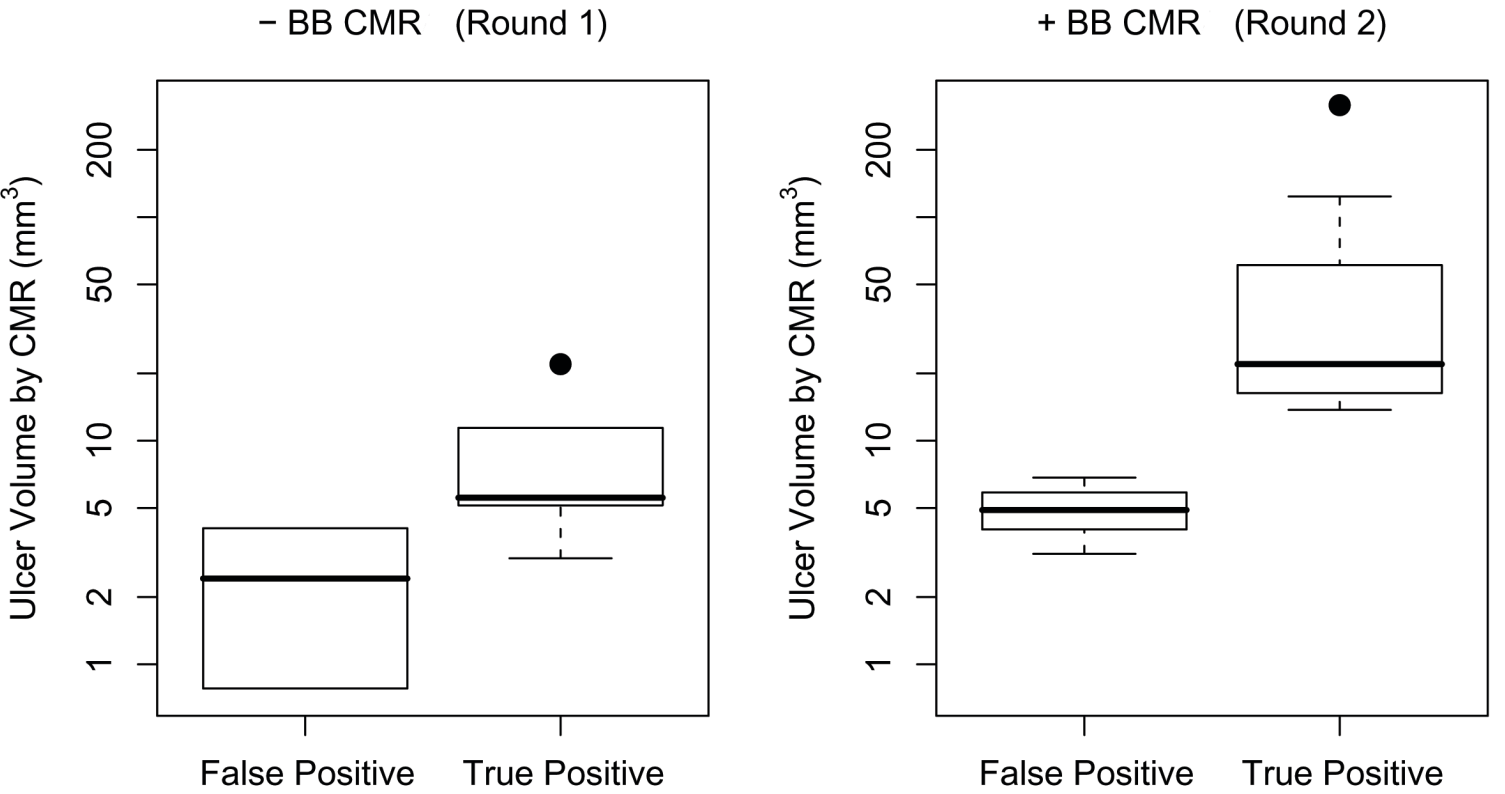
**Distributions of CMR ulcer volume measurements, grouped by whether they corresponded to true or false positives**. The smaller CMR ulcer volume measurements tended to be false positives, especially in round 2 [p = 0.19 for round 1 (cross-sectional CMR images alone, and p = 0.005 for round 2 (cross-sectional CMR images plus BB CMR angiography).

In round 2, the true positive ulcers by CMR had larger volumes than the false positives (median 22 mm^3 ^vs. 4.9 mm^3^, p = 0.005) (Figure 6). In particular, the three false positives were all less than 7 mm^3 ^by CMR whereas the true positives were all greater. 100% specificity could have been achieved without sacrificing sensitivity simply by discarding those CMR measurements. A similar result, though not significant (p = 0.19), was seen in round 1.

## Discussion

In this study, the improvement of ulcer identification can be attributed to the combination of cross-sectional imaging, which has greater resolution in the axial plane but two millimeter slice thickness, and longitudinal BB CMR angiography, which provides higher resolution images along the long-axis in the longitudinal direction. This combination improves the ability to identify carotid plaque ulceration by reducing errors in interpretation caused by mis-registration due to partial volume effects (Figure 3) [23-25]. On the other hand, the addition of the longitudinal BB CMR angiography did result in increased false positives. However, since both the positive and negative predictive values increased modestly, higher sensitivity represents improvement by the addition of the longitudinal BB CMR in the ulcer identification.

We also demonstrate that the sensitivity of ulcer detection depends on the size of ulcers. When longitudinal BB CMR angiography was added to the cross-sectional CMR images, the sensitivity was 80% when only ulcers with volume at least 6 mm^3 ^were evaluated compared to a sensitivity of 52.4% for all ulcers. There were no ulcers between 3.5 and 6 mm^3 ^(Figure 5) and the overall sample size was small, so the threshold of 6 mm^3 ^could not be estimated precisely. We believe that the dependence of sensitivity on ulcer size is related to the spatial resolution provided by the cross-section CMR images and longitudinal BB CMR angiography. As 3.5–6 mm^3 ^corresponds to an average of 2.4–2.9 pixels in each of the three dimensions of cross-sectional CMR images and BB CMR angiography (0.625 mm/pixel with 2 mm thickness for each 2D image), our results suggest the minimum requirement of spatial resolution of CMR for consistent ulcer identification is at least 2–3 pixels.

Low sensitivity occurred in the first round of review with cross-sectional CMR images alone. This can be attributed to two factors. First, presence of partial volume effect and/or flow artifact in the cross-sectional images only led to more false negatives, which subsequently caused lower sensitivity (Figure 3). Second, a conservative approach was used for identifying ulcers by CMR, i.e. ulcers were only identified when the reviewers were very confident of the assessment.

Drawing direct comparisons between the sensitivity and specificity in this study with others is difficult because different imaging modalities and different postprocessing methods were used for ulcer detection [9,31-34]. For ulcer detection, conventional angiography generated a sensitivity of 46% and a specificity of 74%, as it used limited views (e.g., anterior-posterior/oblique/lateral acquisitions) in the NASCET trial [9]. Single-slice CTA resulted in a sensitivity of 60% and a specificity of 74% [31]. Multi-detector CTA showed a sensitivity of 94% and a specificity of 99% [32]. A combination of axial images, maximum intensity projection, multi-planar reformatting, and volume rendering on Multi-detector CTA resulted in higher sensitivity (94%) and specificity (99%) [34]. Therefore, influence of imaging techniques and post processing methods should be taken into account when comparing the accuracy of ulcer detection.

Furthermore, direct comparison between studies of ulceration is difficult because definitions of ulceration vary both among imaging modalities and in histology [9,31-33]. In imaging modalities, ulceration is defined by its shape (crater penetrating into a stenotic plaque or double density on ''en face'' view), its relationship to the vessel lumen (extension of contrast beyond the vascular lumen into surrounding plaque), or irregularity of luminal surface (break in the surface of the plaque with a depth of ≥1 mm) [9,31-33]. In histology, ulceration is defined by the integrity of the plaque surface (disruption in the lining of the plaque or a pit and depression in the plaque, fissuring of the fibrous cap, or break in the surface of the plaque), and/or by penetration depth (approximately ≥1 mm or ≥2 mm) [9,31-33].

In this study, 53% (17 of 32) plaques examined by histology had ulcers. This is consistent with the prevalence of ulceration reported in other studies [35,36]. Park et al reported that 77% of carotid lesions removed from symptomatic patients (transient ischemic attack, amaurosis fugax, prior stroke) and 60% of plaques removed from asymptomatic patients with high-grade stenosis had ulceration [35]. Saam et al reported 25 of 46 (54%) carotid plaques had fibrous cap rupture [36]. All these patients have similar demographic characteristics as our patients in this study. Therefore, the prevalence of ulceration reported in this study is representative of typical demographic characteristics in a population of subjects who had experienced a transient ischemic attack, amaurosis fugax, or stroke within 6 months of their surgery and had >50% carotid stenosis or who were asymptomatic with >80% carotid stenosis by duplex ultrasound.

Our study had several limitations. Longitudinal BB CMR angiography is acquired by a 2D multi-slice double inversion recovery sequence through the centers of internal and external carotid arteries at the carotid bifurcation. If the artery is tortuous, not all segments of the carotid can be covered by this sequence, therefore, ulceration may not be well identified. Implementation of 3D isotropic resolution carotid wall CMR may improve visualization of all segments of carotid artery and allow for better identification of ulceration [37]. In addition, BB CMR angiography may still be insufficient for cases where flow artifact confounds ulcer identification. Use of a motion sensitized driven equilibrium (MSDE) turbo spin echo sequence on 3T CMR may assist in suppression of plaque-mimicking flow artifacts and improve image quality for high-resolution black-blood imaging of carotid arteries [38,39]. This sequence has demonstrated promise in the identification of carotid atherosclerotic ulceration [38]. Furthermore, use of 8-element phased array carotid coils will improve over 4-element coils in the longitudinal coverage and vessel wall signal-to-noise ratio and contrast-to-noise ratio [40]. The number of patients examined is relatively small. Due to the limited number of subjects included, accuracy of adding longitudinal BB CMR angiography needs to be further confirmed by including larger number of subjects in a future study. In addition, due to the small number of subjects, we did not correlate the presence and sizes of ulceration with neurological events in this study. Finally, the study was not blinded.

## Conclusion

This study demonstrates that adding longitudinal black-blood CMR Angiography to multisequence high-spatial-resolution cross-sectional CMR images improves identification of carotid atherosclerotic ulcerations.

## Competing interests

The authors declare that they have no competing interests.

## Authors' contributions

WY performed CMR image review and collection of data. She also contributed to the drafting of the manuscript. HRU performed CMR image peer review. He contributed significantly to the statistical interpretation of data, study design, and revision of the manuscript. MSF was in charge of histological data analysis and revision of the manuscript. DSH collected and analyzed data, performed statistical analysis, prepared figures, and revised the manuscript. CY is a principal investigator of this study's grant. He also assisted in data interpretation and manuscript revision. TSH assisted in data interpretation and was key to revising the critical content of the manuscript. BC participated in CMR image acquisitions, was involved with study design, and was in charge of manuscript preparation and revision of the manuscript. All authors have read and approved submission of this manuscript. The material in the manuscript has not been published and is not being considered for publication elsewhere in whole or in part in any language.
